# Balancing Oncologic Safety and Fertility: A Case of Borderline Ovarian Tumor

**DOI:** 10.7759/cureus.109745

**Published:** 2026-05-27

**Authors:** Elene Lipartia, Beka Metreveli, Natia Marghishvili, David Gagua, Tinatin Gagua

**Affiliations:** 1 Obstetrics and Gynecology, Gagua Clinic, Tbilisi, GEO; 2 Obstetrics and Gynecology, David Tvildiani Medical University, Gagua Clinic, Tbilisi, GEO

**Keywords:** fertility-sparing surgery, frozen section, multi-disciplinary teams, omentectomy, ovarian neoplasm, patient-centered care, permanent section, serous borderline tumor

## Abstract

Borderline ovarian tumors (BOTs) are epithelial neoplasms that mostly affect women of reproductive age; management requires a balance between oncological safety and fertility preservation. The aim of this case is to illustrate the importance of intraoperative decision-making, patient-centered care, and fertility-sparing management.

We present a case of a 31-year-old woman whose right ovarian mass was initially diagnosed as a dermoid cyst by ultrasound. Intraoperatively, a borderline tumor was suspected, and biopsy of both ovaries, omental sampling, and peritoneal lavage for cytology were done. Histopathological examination confirmed a borderline tumor of serous type in the right ovary and a corpus luteum cyst in the left ovary.

In the subsequent stage, a right salpingo-oophorectomy, a repeat biopsy of the left ovary, pelvic lavage, omentectomy, and appendectomy were performed using a laparoscopic approach. This case highlights the importance of individualized, stepwise surgical management, ensuring both oncological safety and fertility preservation.

## Introduction

Borderline ovarian tumors (BOTs) represent a heterogeneous group of pathologies characterized histologically by proliferation of atypical epithelial cells without evidence of stromal invasion; their biological features are markedly different from those of low-grade ovarian carcinoma, which is why they are considered an independent clinical entity [1]. BOTs account for approximately 15% of epithelial ovarian tumors and were first described in the literature over a century ago. Histologically, they are divided into mucinous (MBOTs), serous (SBOTs), endometrioid, clear cell, Brenner type, and other. Most of these tumors are confined to one or both ovaries, and approximately 75% of cases are diagnosed at an early stage [2,3].

BOTs often present clinically like other accessory masses; some patients are asymptomatic, while others may present with pelvic or abdominal pain, pressure, or dyspareunia [1]. Most of the cases are diagnosed by ultrasound, which, when performed by an experienced sonographer, distinguishes benign from malignant masses with high accuracy. Borderline tumors are often discovered incidentally in asymptomatic patients as ovarian cysts with internal papillary formations [4]. The diagnostic evaluation is similar to ovarian carcinoma, although serum CA-125 is not a reliable marker. In approximately one-third of cases, the diagnosis must be revised using permanent sections (PSs). Frozen section (FS) studies are largely directed at the benign assessment of borderline tumors and, much less frequently, at the overdiagnosis of carcinoma. It is noteworthy that approximately 20%-30% of specimens identified as borderline tumors by FS are classified as carcinoma upon subsequent PS review, especially in the mucinous type (33%) and serous type (13%). Only rarely, approximately 5%, is a FS borderline ovarian tumor diagnosis revised to benign on the basis of PSs [4].

BOTs are staged according to the International Federation of Gynaecology and Obstetrics (FIGO) and the American Joint Committee on Cancer (AJCC), most commonly according to the FIGO system [5,6]. According to current guidelines (FIGO and AJCC classification), the mainstay of treatment for BOTs is surgery, while in early stages (stage I or II), fertility-preserving surgery is possible if it is oncologically safe; for advanced disease (stage III or IV), treatment is total hysterectomy, removal of both ovaries and fallopian tubes, omentectomy, lymph node dissection, and optimal cytoreduction [5,7]. Survival rates are highest in patients with no macroscopic residual tumor after surgery and are significantly lower in patients with residual disease [7]. In completely resected cases, additional adjuvant therapy is usually not required. The overall prognosis is favorable, although individual surgical planning is important to maintain a balance between fertility preservation and oncological safety.

This work describes the case of a 31-year-old woman with a confirmed FIGO-classified BOT. The report is prepared in a standard surgical case format and aims to highlight the importance of balancing fertility preservation and oncological safety.

## Case presentation

**Figure 1:**
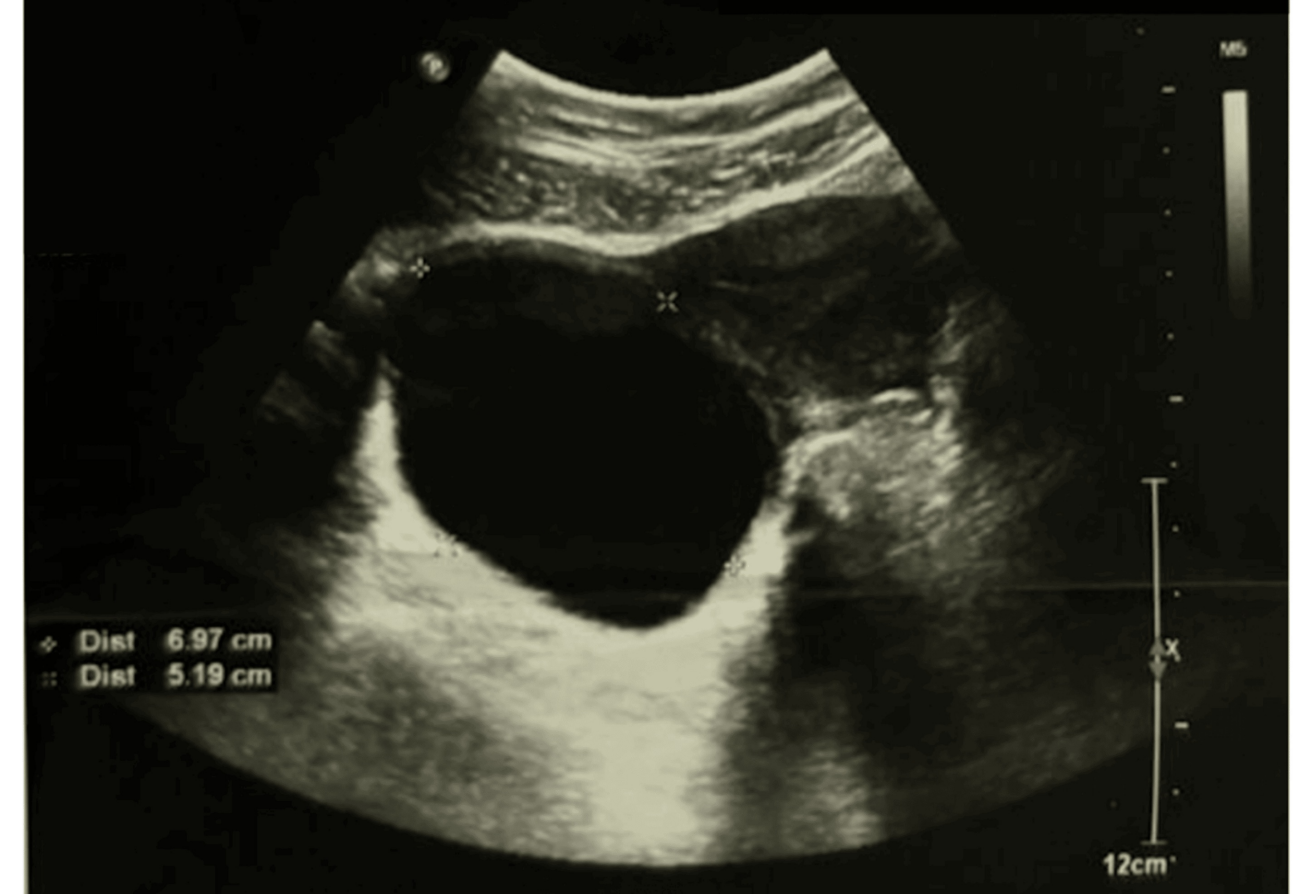
Pelvic ultrasound The right ovary with hyperechoic inclusions with clearly contoured anechoic formations, measuring 70 x 62 x 79 mm. Normal follicles are identified. No free pelvic fluid is seen in the cul-de-sac.

We present a 31-year-old G0P0 woman with right pelvic fullness and intermittent hypogastric pain, mostly right-sided. Secondary amenorrhea was observed for the last six months. Gynecological ultrasound revealed the right ovary with hyperechoic inclusions with clearly contoured anechoic formations, measuring 70 x 62 x 79 mm, suggesting a dermoid cyst (Figure 1). The uterus appeared normal in size and echogenicity, with a normal endometrial stripe (10 mm) and preserved vascular flow. The left ovary showed normal morphology with preserved vascularity and visible follicles. No free fluid was detected in the pelvis. Laboratory data were generally within normal limits, except for a mild increase in CA-125 levels (57.9 IU/mL, normal range 0.0-35.0 IU/mL). Infectious screening was negative; coagulation parameters and complete blood count were unremarkable (Table 1).

**Table 1:** Laboratory findings include infectious diseases (HBsAg, anti-HCV, HIV 1/2 Ag/Ab, and syphilis TP), complete blood count, coagulation parameters, and CA-125 HBsAg: hepatitis B surface antigen; HCV: hepatitis C virus; Ag/Ab: antigen/antibody; TP: *Treponema pallidum*; PT: prothrombin time; PI: prothrombin index; INR: international normalized ratio; PTT: partial thromboplastin time; MCV: mean corpuscular volume; MCH: mean corpuscular hemoglobin; MCHC: mean corpuscular hemoglobin concentration; RDW-CV: red cell distribution width-coefficient of variation; MPV: mean platelet volume; PDW: platelet distribution width; PCT: plateletcrit

Test	Result	Reference range	Unit
HBsAg (hepatitis B)	Negative	Negative	-
Anti-HCV (hepatitis C)	Negative	Negative	-
HIV 1/2 Ag/Ab	Non-reactive	Non-reactive	-
Syphilis TP	Non-reactive	Non-reactive	-
Glucose	83.20	55.0-115.0	mg/dL
PT	12.10	9.5-13.0	sec
PI	83.00	70.0-115.0	%
INR	1.15	0.9-1.2	-
PTT	28.5	25.0-36.5	sec
Fibrinogen	337.00	240.0-450.0	mg/dL
CA-125	57.90	0.0-35.0	IU/mL
White blood cells (WBC)	7.82	4.0-10.0	×10³/µL
Red blood cells (RBC)	4.07	3.9-5.2	×10⁶/µL
Hemoglobin (Hgb)	12.34	12.0-15.5	g/dL
Hematocrit (HCT)	35.00	33.0-49.0	%
MCV	86.00	80.0-95.0	fL
MCH	30.30	26.0-34.0	pg
MCHC	35.30	31.0-35.0	g/dL
RDW-CV	12.20	10.0-17.0	%
Platelets (PLT)	249.30	150-450	×10³/µL
MPV	8.30	7.0-11.0	fL
PDW	15.90	15.0-18.0	%
PCT	0.21	0.16-0.40	%

**Figure 2:**
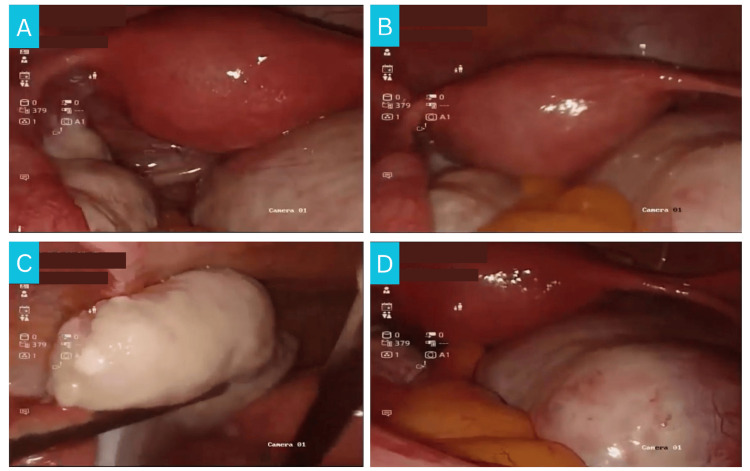
Intraoperative laparoscopic findings (A, B) A normal-sized uterus with grossly normal bilateral fallopian tubes. (C) Left ovary with film-hard consistency formation. (D) Enlarged right ovary.

The patient was scheduled for laparoscopic (LS) excision of the right ovarian cyst. Intraoperatively, the uterus was of normal size, dense, and smooth-edged. Both fallopian tubes were without pathology. The right ovary was enlarged (10 × 6 cm) due to the formation; on the medial and lateral surfaces, papillary inclusions were noted. Formation of a hard consistency was detected on the left ovary (Figures 2A-2D). Intraoperatively, the nature of the tumor led to the suspicion of an ovarian neoplasm, based on which a consultation was convened. According to the decision made, the procedure was limited to staging and diagnostic interventions. With the written consent of the patient's family, right and left ovarian biopsies, omental tissue samples, and peritoneal lavage were obtained for cytological and histological examination.

**Figure 3:**
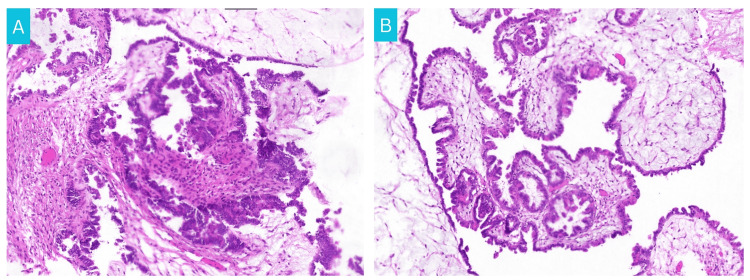
Microscopic view of the right ovary (A, B) Tissue sample taken from the right ovary shows a papillomatous formation with a markedly swollen, hemorrhagic-hyalinized stroma, covered with cuboidal epithelium with disrupted nuclei and broadly eosinophilic, apical processes. In some areas, the papillary structures are characterized by hierarchical branching, and invaginated glandular structures are also seen in the stroma.

**Figure 4:**
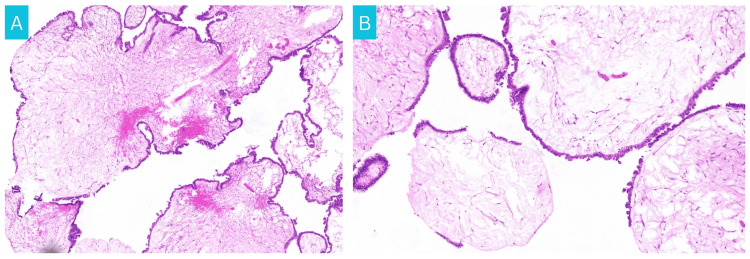
Microscopic view of the left ovary (A, B) Tissue samples taken from the ovarian stromal component containing follicular structures, in an area where cyst samples are revealed, lined with a granulosa cell layer with multilayered eosinophilic cytoplasm.

The peritoneal lavage revealed a moderate number of lymphocytes and a small number of reactive mesotheliocytes, without cellular atypia and reactive changes in the peritoneum. Histopathological analysis showed a serous borderline tumor of the right ovary and left ovarian corpus luteum cyst (Figures 3A, 3B, 4A, 4B).

**Figure 5:**
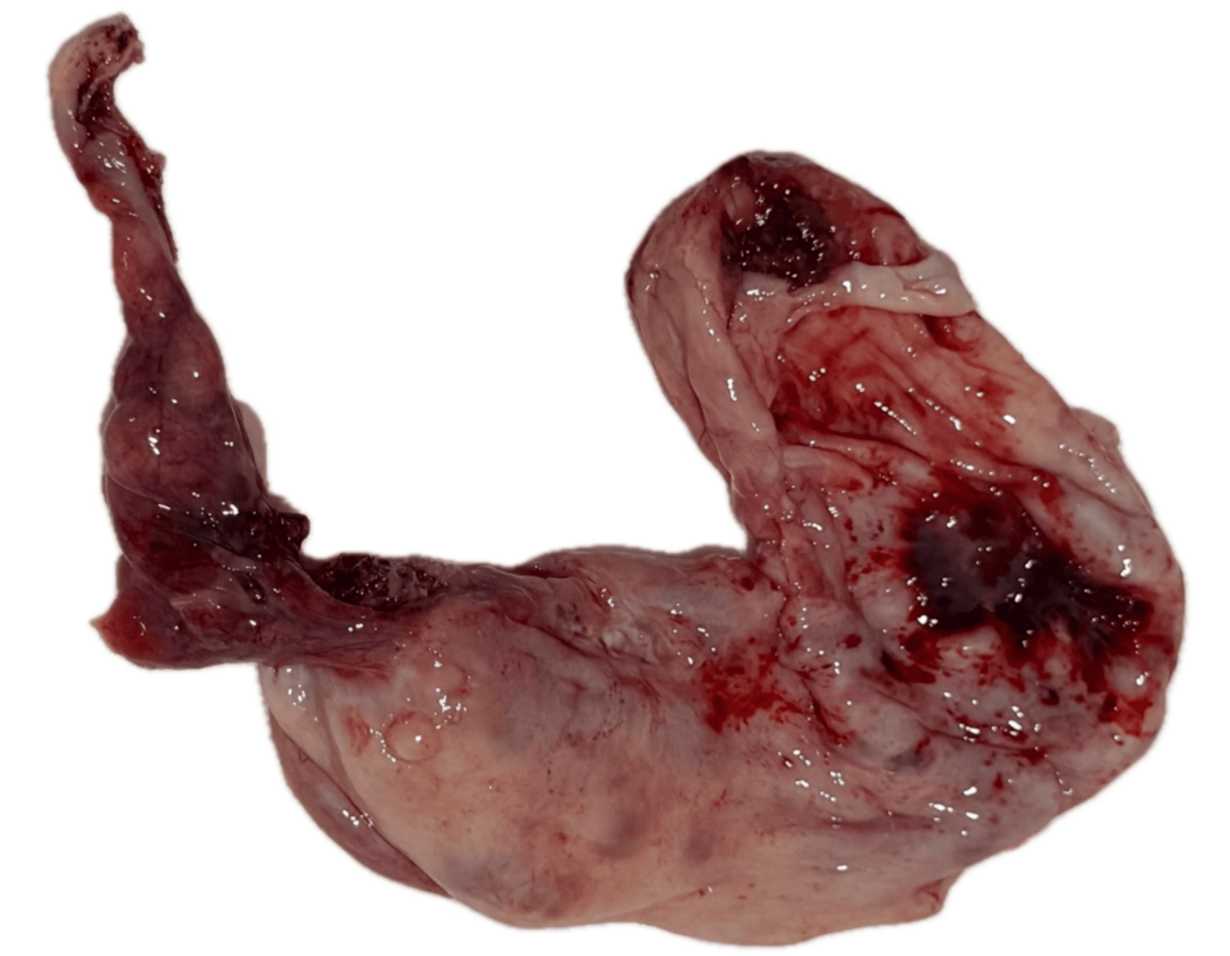
Gross specimen of the resected tissues of the right fallopian tube and ovary with associated adnexal structures

**Figure 6:**
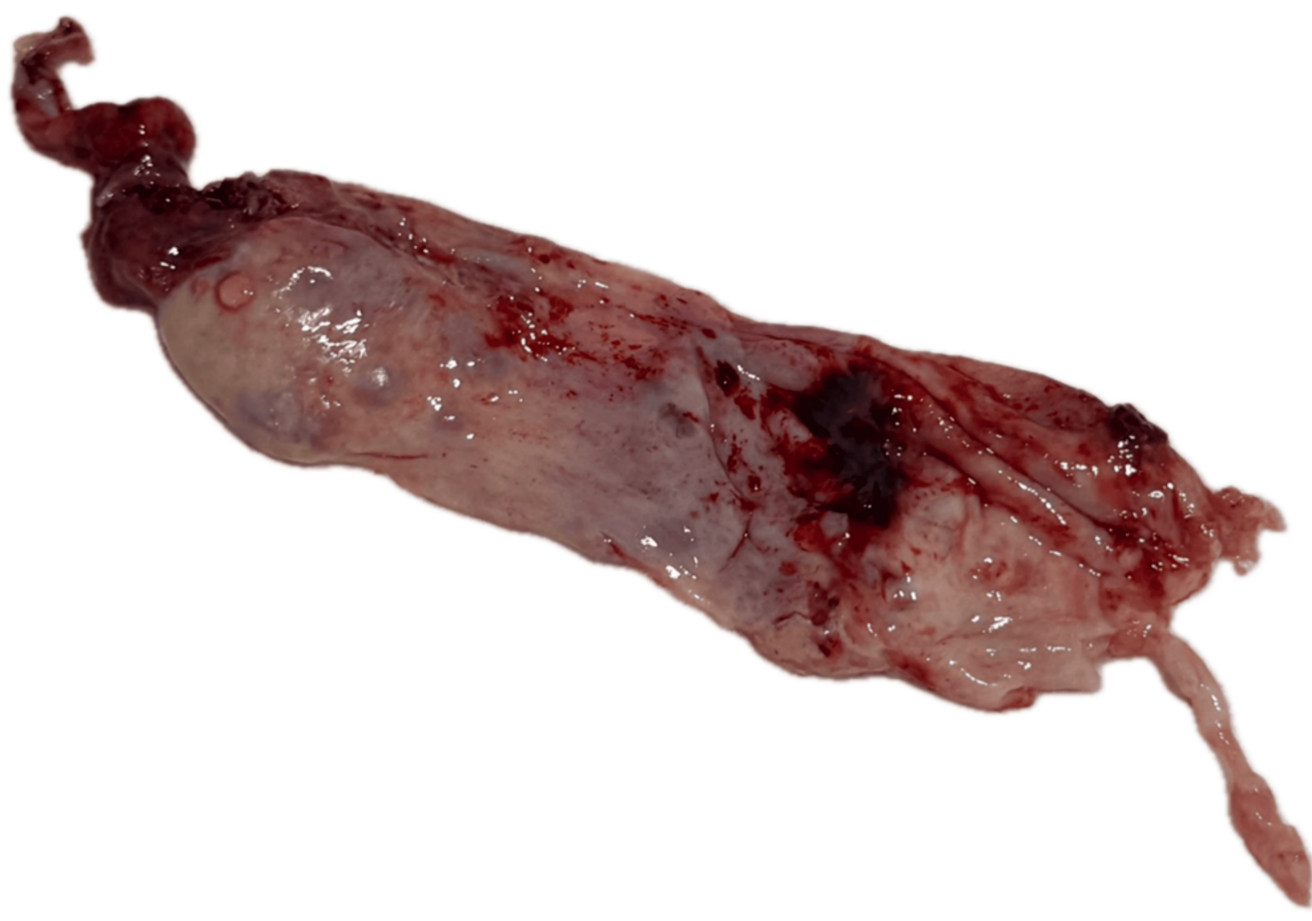
Gross specimen of the salpingo-oophorectomy specimen, with visible papillary excrescences

**Figure 7:**
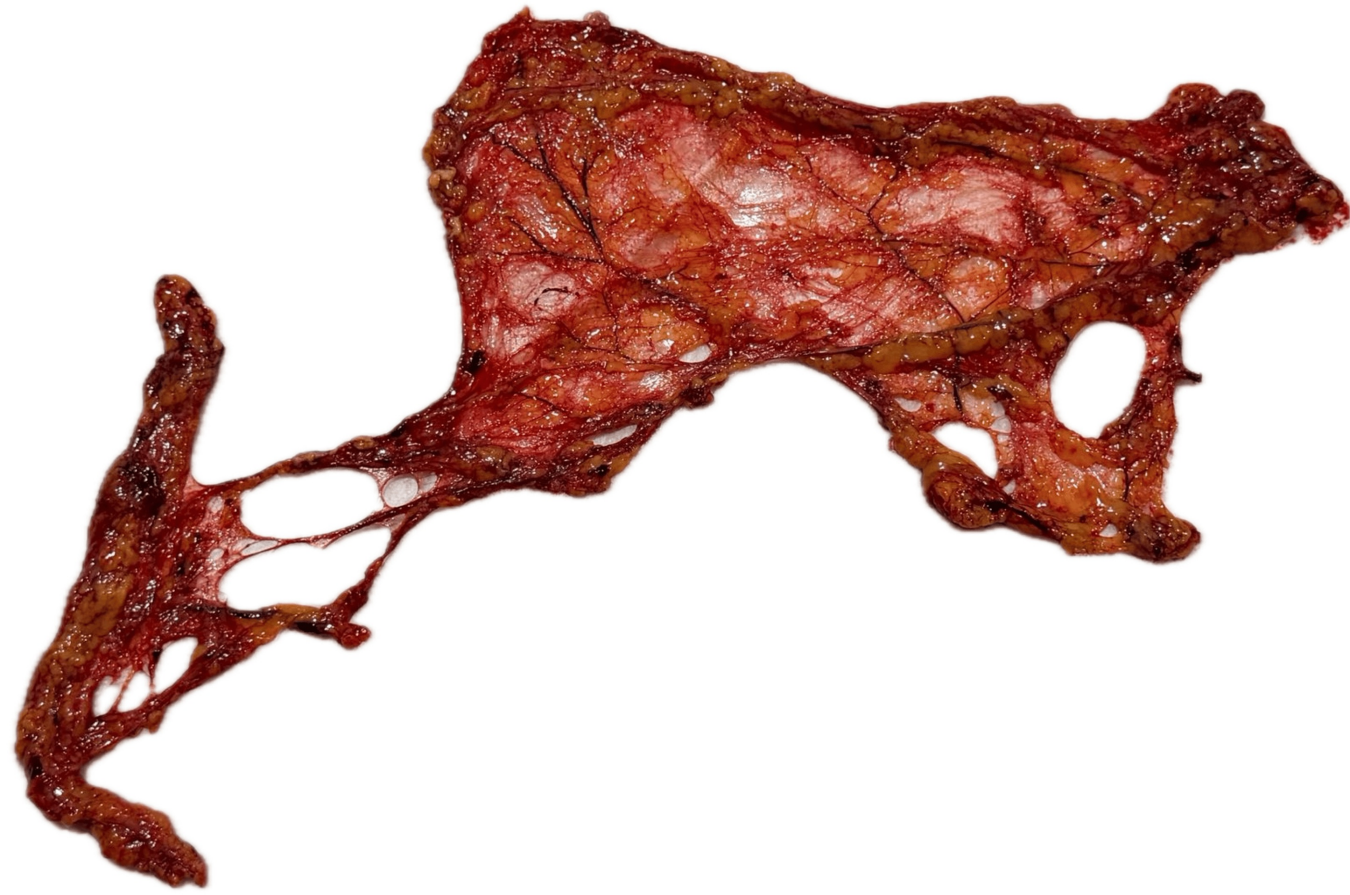
Macroscopic view of the resected omentum

**Figure 8:**
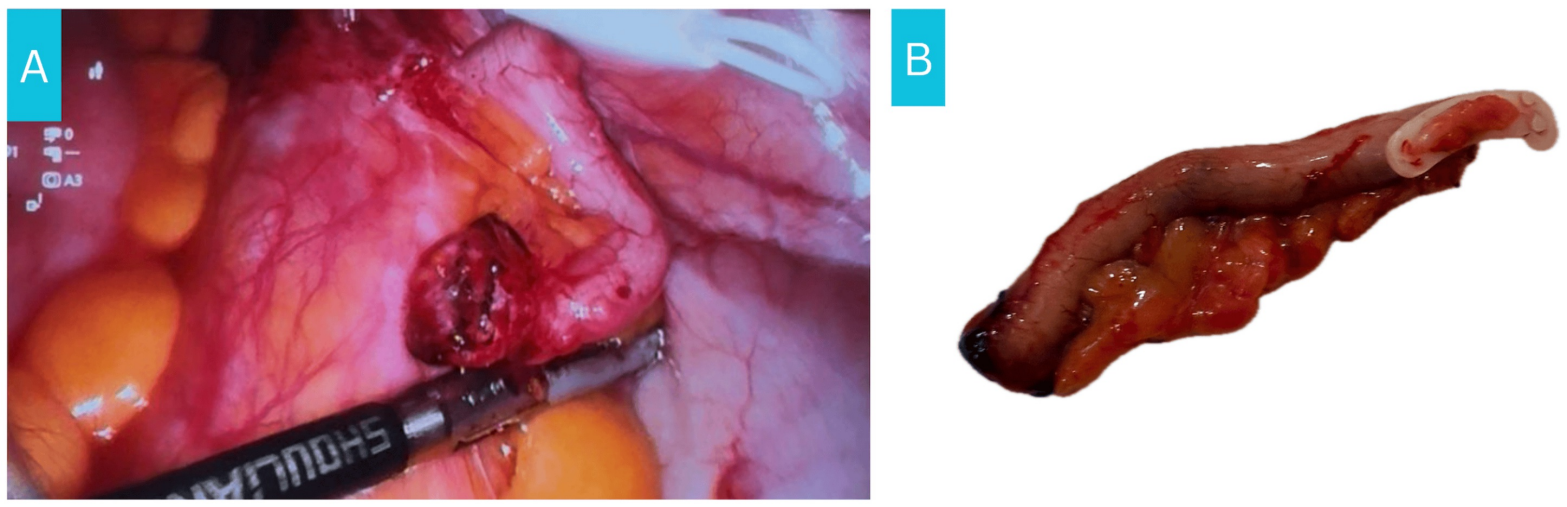
Macroscopic view of the appendix (A) Intraoperative laparoscopic view showing active bleeding from the appendix. (B) Resected specimen of the appendix.

The second surgical intervention was planned. The patient is 31 years old (G0P0), and preserving reproductive function was a priority for her, which is why the decision was made by a multidisciplinary team - Reproductologist, Gynecologist, and Oncogynecologist. A laparoscopic operation was performed, during which right adnexectomy with the mass (Figures 5, 6), omentectomy (Figure 7), peritoneal washing for cytological examination, and left ovarian biopsy were done. Intraoperatively, the right ovary was found to be attached to the appendix apex. During the mobilization process, the tip of the appendix was bleeding, which is why an appendectomy was performed (Figures 8A, 8B).

The resected mass was placed in an endobag, after which an instrument was introduced into the bag through the trocar insertion point. The mass contents were aspirated to reduce its size; after, the material was removed through the endobag, with full containment. All specimens taken during the operation were sent for histological and cytological examination to establish a final diagnosis.

Serous borderline tumor of the right ovary

**Figure 9:**
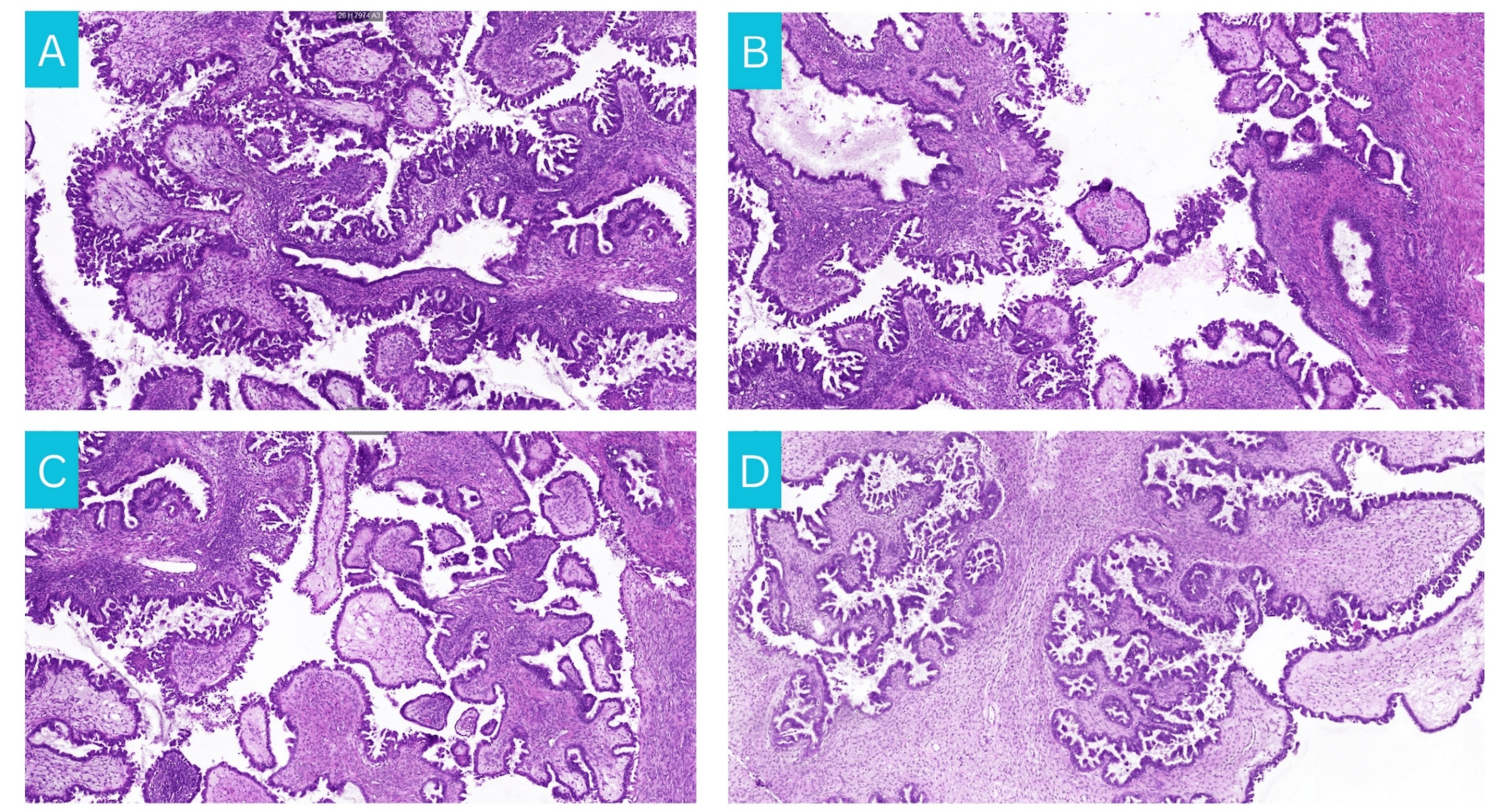
Microscopic view of the right ovary (A-D) In tissue samples from the right ovary with intact capsule, foci with fibrous-hyaline connective tissue stroma lined with ciliated cylindrical epithelium are revealed in the lumens of cystic structures, with focal formation of broad-axial macropapillary structures, accompanied by psammomatous calcifications. The cells contain granular chromatin nuclei with pronounced nucleoli and mitotic figures.

The tumor is localized to the ovary, without capsule or surface invasion. The surface of the fallopian tube is intact. No peritoneal implants are evident. No atypical cells are detected in the peritoneal fluid. The final pathological stage based on the eighth edition of the American Joint Committee on Cancer was pT1a pNx pMx, and according to FIGO stage: IA (Figures 9A-9D) [5,6].

## Discussion

Mostly, BOTs are diagnosed at an early stage, which significantly improves survival rates. According to the literature, five-year survival in early stages is more than 95% [1,8]. The prognosis is influenced by the stage of the disease, the presence of peritoneal implants, residual disease after surgery, and histological type. The gold standard for the management of BOT is surgical treatment. The disease is asymptomatic in approximately 30% of cases; 50%-60% of patients present with symptoms such as abdominal pain or distension. Most BOTs are identified by transvaginal ultrasonography; it is widely accepted as a preoperative method for differentiating benign from malignant adnexal masses, especially when performed by an experienced specialist. Ultrasound can detect peritoneal implants with high accuracy (91%-95%) and provides important information for preoperative planning and staging [1]. If suspicion remains after ultrasound, MRI should be performed, as it is the standard imaging modality for the evaluation of adnexal masses that are not visualized by ultrasound [9,10]. The term “at least borderline” or similar terminology is often used, suggesting the possibility of invasive carcinoma [11]. A retrospective study of 222 ovarian biopsies evaluated the diagnostic accuracy of FS. The sensitivity for benign, malignant, and borderline tumors was 98%, 88.7%, and 61% [12]. Surgical staging procedures include abdominal inspection, omentectomy, peritoneal biopsies, and peritoneal lavage fluid examination. Surgical treatment options are hysterectomy, fertility-sparing surgery like ovarian cystectomy or unilateral salpingo-oophorectomy, and bilateral salpingo-oophorectomy [13,14]. Restaging of BOTs is necessary in cases where cystectomy has been performed and histology shows micropapillary or cribriform structures, also when a complete abdominal and pelvic evaluation was not performed at the initial operation.

Treatment for BOT is surgical management, with the goal of complete staging and removal of macroscopic disease [7,15]. In FIGO stage I serous-type BOT, surgical treatment is sufficient in most cases, and adjuvant therapy is not recommended [7,15]. In our case, a stepwise surgical management strategy was chosen for a young 31-year-old patient. The first stage included diagnostic laparoscopy with biopsy, and the second stage included definitive treatment with staging surgery. A similar two-step approach has been described in the literature as an acceptable strategy in cases where staging is required and the extent of surgery needs to be individualized [15,16]. One of the controversial issues in the surgical management of BOT is the choice of laparoscopy or laparotomy. Historically, laparotomy was preferred due to its oncological safety, but recent data show that in properly selected patients, the laparoscopic approach does not worsen oncological outcomes [13,17]. However, laparoscopy may be associated with a relatively high risk of intraoperative tumor damage, which requires special technical caution [16]. In our case, special attention was paid to oncological principles, and the tumor was removed using an endobag, which plays an important role in preventing peritoneal contamination. Importantly, there was no rupture of the tumor into the abdominal cavity, which is considered a positive prognostic factor, as intraperitoneal spillage may be associated with an increased risk of recurrence [17]. According to the current guidelines of the European Society of Gynaecological Oncology (ESGO) and the European Society of Medical Oncology (ESMO), fertility-sparing surgery is recommended for young patients with FIGO stage I serous BOT [14-16]. In such cases, unilateral salpingo-oophorectomy is preferred over cystectomy, if possible, because cystectomy has a relatively high risk of recurrence. Systemic lymph node dissection is not recommended unless clinically or intraoperatively suspicious lymph nodes are identified, as it does not improve survival rates [15,16]. Regarding the guidelines of ESMO and ESGO, appendectomy is not routinely considered mandatory in serous BOT, but in the case of a macroscopically pathological appendix, its resection is recommended as part of staging [15,16].

An example of Mohapatra et al. demonstrated that sparing surgery is mostly effective in young patients, and the final diagnosis still depends on detailed histopathological analysis. Review of the literature describes that FS cannot provide reliable differentiation between borderline and malignant processes, which clinically significantly increases the risk of incorrect intraoperative decisions [18]. This problem is not isolated and reflects broader systemic limitations. The same view is supported by a systematic review by Ferrero et al., which indicates that the inconsistent accuracy of FS leads to a substantial rate of both underdiagnosis and overdiagnosis [19]. In our case, the contralateral ovary, fallopian tube, and uterus were preserved, considering the patient's reproductive plans. According to available data, although conservative surgery is associated with a relatively high recurrence rate (approximately 10%-20%), the overall survival is not different from radical surgery [17,20]. Our approach reduces the risk of missing synchronous pathology or occult processes. Omentectomy is also an important component of staging, as microscopic implants can be present, despite their low frequency [20]. The risk of recurrence in BOT varies from 5% to 30% according to the literature and depends on the type of surgery, stage, and histological features [17,20]. Recurrences are more common after conservative surgery, although in most cases they are still borderline and can be successfully treated with repeat surgery [20].

## Conclusions

The management of suspicious ovarian masses requires a strictly staged, individualized, and evidence-based surgical approach. In case of diagnostic uncertainty, minimally invasive initial intervention is recommended to avoid overtreatment. Long-term follow-up in this patient group is critically important, since in the case of BOT FIGO early-stage disease, the risk of late recurrence cannot be entirely excluded.

Intraoperative FS examination is an important tool, but its limited sensitivity and specificity are well-documented and may lead to misdiagnosis of surgical treatment. Therefore, surgical decisions based solely on this method are not recommended. Definitive treatment strategies should be based on the final histopathological diagnosis and should be implemented in strict accordance with oncological principles, with informed consent of the patient. In selected cases, particularly FIGO stage IA borderline serous ovarian tumors, fertility-sparing surgery is an oncologically acceptable and safe approach in patients of reproductive age. This case highlights that BOTs require a multidisciplinary, evidence-based, and patient-centered approach that represents the optimal balance between oncological safety and preservation of reproductive function.

## References

[1] Chen LM, Berek JS (2026). Borderline ovarian tumors. UpToDate.

[2] Gaballa K, Abdelkhalek M, Fathi A, Refky B, Belal K, Elaraby M, Zuhdy M (2022). Management of borderline ovarian tumors: a tertiary referral center experience in Egypt. Front Surg.

[3] Della Corte L, Cafasso V, Mercorio A, Vizzielli G, Conte C, Giampaolino P (2023). Editorial: management of borderline ovarian tumor: the best treatment is a real challenge in the era of precision medicine. Front Surg.

[4] du Bois A, Trillsch F, Mahner S, Heitz F, Harter P (2016). Management of borderline ovarian tumors. Ann Oncol.

[5] Wilailak S, Berek JS (2025). FIGO Cancer Report 2025: transforming gynecologic oncology through global equity, technological innovation, and preventive strategies. Int J Gynaecol Obstet.

[6] Amin MB, Greene FL, Edge SB (2017). The Eighth Edition AJCC Cancer Staging Manual: continuing to build a bridge from a population-based to a more "personalized" approach to cancer staging. CA Cancer J Clin.

[7] PDQ® Adult Treatment Editorial Board (2026). PDQ Ovarian Borderline Tumors Treatment. https://www.cancer.gov/types/ovarian/hp/ovarian-borderline-tumors-treatment-pdq.

[8] Baker‐Rand H, Bolton J, Morgan RD, Edmondson R (2025). Borderline ovarian tumours. Obstet Gynecol.

[9] Tsuboyama T, Sato K, Ota T (2022). MRI of borderline epithelial ovarian tumors: pathologic correlation and diagnostic challenges. Radiographics.

[10] Li K, Song F, Yu L, Shi H, Wang J, Cheng X (2022). Role of MRI in characterizing serous borderline ovarian tumor and its subtypes: correlation of MRI features with clinicopathological characteristics. Eur J Radiol.

[11] De Decker K, Jaroch KH, Edens MA (2021). Frozen section diagnosis of borderline ovarian tumors with suspicious features of invasive cancer is a devil's dilemma for the surgeon: a systematic review and meta-analysis. Acta Obstet Gynecol Scand.

[12] Gol M, Baloglu A, Yigit S, Dogan M, Aydin C, Yensel U (2003). Accuracy of frozen section diagnosis in ovarian tumors: is there a change in the course of time?. Int J Gynecol Cancer.

[13] Fauvet R, Boccara J, Dufournet C, Poncelet C, Daraï E (2005). Laparoscopic management of borderline ovarian tumors: results of a French multicenter study. Ann Oncol.

[14] Kasaven LS, Chawla M, Jones BP (2022). Fertility sparing surgery and borderline ovarian tumours. Cancers (Basel).

[15] Ledermann JA, Matias-Guiu X, Amant F (2024). ESGO-ESMO-ESP consensus conference recommendations on ovarian cancer: pathology and molecular biology and early, advanced and recurrent disease. Ann Oncol.

[16] Fischerova D, Zikan M, Dundr P, Cibula D (2012). Diagnosis, treatment, and follow-up of borderline ovarian tumors. Oncologist.

[17] Seong SJ, Kim DH, Kim MK, Song T (2015). Controversies in borderline ovarian tumors. J Gynecol Oncol.

[18] Mohapatra I, Samantaray SR, Harshini N (2022). Fertility-preserving surgery of borderline serous ovarian tumors: a case report. Cureus.

[19] Ferrero S, Morotti M, Venturini PL, Penuela L, Vellone VG, Barra F (2018). Accuracy of intra-operative frozen section in the diagnosis of borderline ovarian tumors and clinical impact of underdiagnosis. Oncol Res Rev.

[20] Vasconcelos I, de Sousa Mendes M (2015). Conservative surgery in ovarian borderline tumours: a meta-analysis with emphasis on recurrence risk. Eur J Cancer.

